# Osimertinib (AZD9291), a Mutant-Selective EGFR Inhibitor, Reverses ABCB1-Mediated Drug Resistance in Cancer Cells

**DOI:** 10.3390/molecules21091236

**Published:** 2016-09-15

**Authors:** Xiao-Yu Zhang, Yun-Kai Zhang, Yi-Jun Wang, Pranav Gupta, Leli Zeng, Megan Xu, Xiu-Qi Wang, Dong-Hua Yang, Zhe-Sheng Chen

**Affiliations:** 1 Department of Pharmaceutical Sciences, College of Pharmacy and Health Sciences, St. John’s University, Queens, NY 11439, USA; xiaoyu.zhang14@my.stjohns.edu (X.-Y.Z.); yunkai.zhang12@stjohns.edu (Y.-K.Z.); yijun.wang11@my.stjohns.edu (Y.-J.W.); pranav.gupta13@my.stjohns.edu (P.G.); zengleli0736@163.com (L.Z.); mxu2@student.gn.k12.ny.us (M.X.); yangd1@stjohns.edu (D.-H.Y.); 2 School of Chemistry and Chemical Engineering, Sun Yat-Sen University, Guangzhou 510275, China; 3 College of Animal Science, South China Agricultural University, Guangzhou 510642, China; xqwang@scau.edu.cn

**Keywords:** osimertinib, multidrug resistance, ABCB1, tyrosine kinase inhibitors

## Abstract

In recent years, tyrosine kinase inhibitors (TKIs) have been shown capable of inhibiting the ATP-binding cassette (ABC) transporter-mediated multidrug resistance (MDR). In this study, we determine whether osimertinib, a novel selective, irreversible EGFR (epidermal growth factor receptor) TKI, could reverse ABC transporter-mediated MDR. The results showed that, at non-toxic concentrations, osimertinib significantly sensitized both ABCB1-transfected and drug-selected cell lines to substrate anticancer drugs colchicine, paclitaxel, and vincristine. Osimertinib significantly increased the accumulation of [^3^H]-paclitaxel in ABCB1 overexpressing cells by blocking the efflux function of ABCB1 transporter. In contrast, no significant alteration in the expression levels and localization pattern of ABCB1 was observed when ABCB1 overexpressing cells were exposed to 0.3 µM osimertinib for 72 h. In addition, ATPase assay showed osimertinib stimulated ABCB1 ATPase activity. Molecular docking and molecular dynamic simulations showed osimertinib has strong and stable interactions at the transmembrane domain of human homology ABCB1. Taken together, our findings suggest that osimertinib, a clinically-approved third-generation EGFR TKI, can reverse ABCB1-mediated MDR, which supports the combination therapy with osimertinib and ABCB1 substrates may potentially be a novel therapeutic stategy in ABCB1-positive drug resistant cancers.

## 1. Introduction

Multidrug resistance (MDR) in cancer, which is defined as the resistance of cancer cells to antineoplastic agents by either intrinsic or acquired mechanisms, is a huge obstacle for successful chemotherapy [1,2]. Many factors, such as efflux transporters, apoptosis regulation and DNA repair, are responsible for the development of MDR, and the most prominent one is associated with the overexpression of membrane ATP-binding cassette (ABC) transporters in cancer cells [3,4,5]. Based on similarities in sequence and structural organization, 49 ABC transporters have been identified and classified into seven subfamilies (A–G), among which ABCB1, AeBCG2, and ABCCs are the primary components in the development of the MDR phenotype [6]. ABCB1, also called P-glycoprotein (P-gp) or multidrug resistance 1 (MDR1), was the first discovered human mammalian ABC transporter since 1976 [7,8]. ABCB1 transporter, which is present in kidney, intestine, placenta, and brain can transport a number of anticancer drugs such as paclitaxel, doxorubicin, and vincristine out of the cancer cells [9,10]. Therefore, modulating the function or expression of ABC transporters, especially ABCB1, has great clinical significance in cancer treatment. There are also several non-ABC-transporter proteins which has been shown to be implicated in MDR, such as lung resistance related protein (LRP), Topoisomerase II α (TopoII α), Annexin A1, Glutathione S-transferase (GST-π), and Ral binding protein (RalBP-1or RLIP76) [11,12]. However, the mechanisms of action and regulation of the non-ABC-transporter proteins are still largely unknown.

An effective way to restore chemosensitivity in MDR cancer cells is to develop inhibitors which either decrease the expression of ABC proteins or inhibit the efflux function of ABC transporters [13]. It has been reported that some tyrosine kinase inhibitors (TKIs), including epidermal growth factor receptor (EGFR) TKIs, significantly reversed ABCB1- and ABCG2-mediated MDR [14]. These TKIs modulate the ATPase activity of ABC transporters and inhibit drug efflux to overcome drug resistance [15,16,17]. Therefore, TKIs play a significant role and may be used as MDR reversal agents in cancer cells.

**Figure 1:**
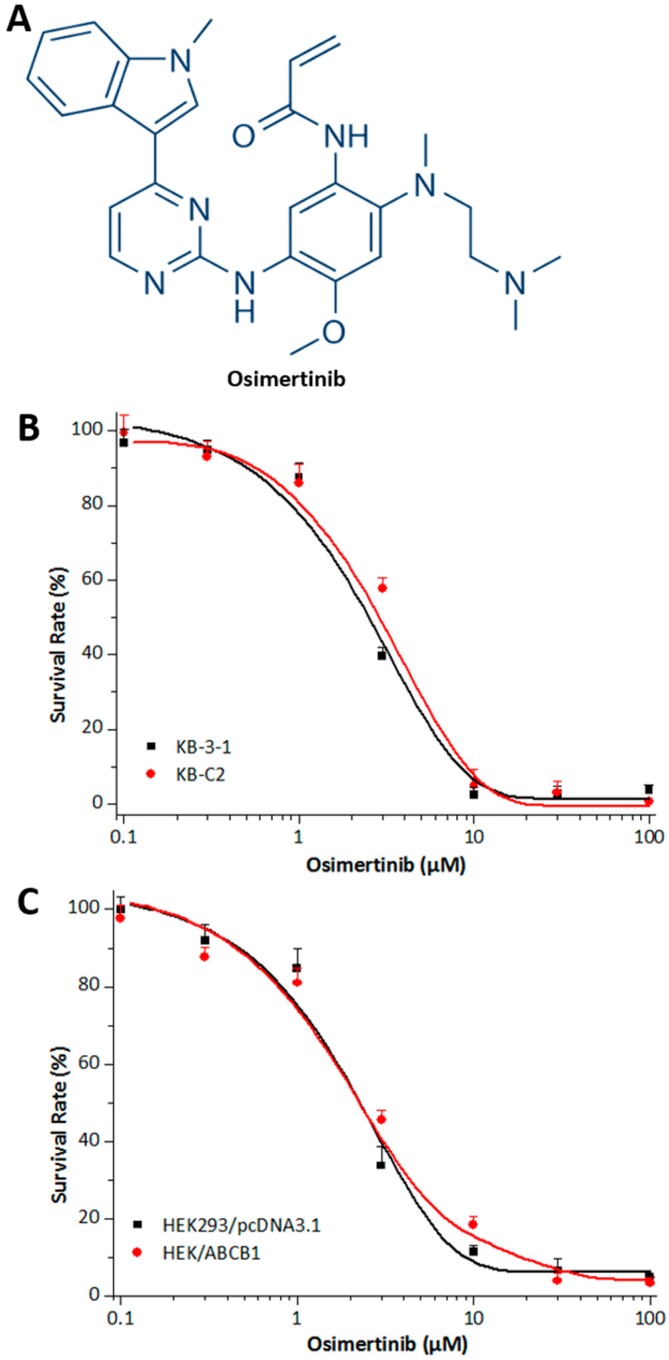
Chemical structure and cytotoxicity of osimertinib in parental and ABCB1 overexpressing cell lines. (**A**) Chemical structure of osimertinib (AZD9291); (**B**) cytotoxicity of osimertinib was determined in KB-3-1 and KB-C2 cell lines; and (**C**) cytotoxicity of osimertinib was determined in HEK293/pcDNA3.1 and HEK/ABCB1 cell lines. Each cell line was incubated with different concentrations of osimertinib for 72 h. Cell survival rate was determined by the MTT assay as described in “Materials and Methods”. Points with error bars represent the mean ± SD.

The first-generation EGFR TKIs, such as erlotinib and gefitinib, and the second-generation EGFR TKIs such as afatinib, have been widely used in the treatment of patients with advanced non-small cell lung cancer (NSCLC) [18,19,20]. However, many patients frequently develop acquired resistance to these inhibitors [21]. The EGFR T790M mutation was present in most of the resistant cases [22]. Recently, the third-generation EGFR inhibitor osimertinib (Figure 1), also called AZD9291 or tagrisso, has emerged as a potential therapeutic to treat patients with metastatic EGFR T790M mutation-positive NSCLC by blocking the growth of EGFR T790M-positive tumors [23,24,25]. Unlike the first- and second-generation EGFR TKIs, the third-generation TKIs have significantly increased the potency for EGFR mutants compared to wild-type EGFR.

In this study, we determine whether osimertinib, a third-generation EGFR TKI which has been approved by the U.S. Food and Drug Administration (FDA) recently, could reverse ABC transporter-mediated multidrug resistance which is associated with the overexpression of ABCB1. Both the first-generation reversal agent verapamil and third-generation reversal agent zosuquidar were used as positive controls in this study.

## 2. Results

### 2.1. Effects of Osimertinib on ABCB1 Substrates in Cell Lines Overexpressing ABCB1

Cytotoxicity assays were performed in order to select a non-toxic drug concentration for osimertinib (Figure 1B,C). Concentrations of 0.3, 0.5, and 1.0 μM at which no significant cytotoxicity were chosen for further experiments. In order to determine the reversal effect of osimertinib on ABCB1-mediated MDR in ABCB1-overexpressing human cancer cells, cell survival assays were performed in the presence and absence of osimertinib, using the parental KB-3-1 cell line and drug-selective KB-C2 cell line. Osimertinib at 1.0 μM did not significantly alter the sensitivity of the parental KB-3-1 cells to the drugs. However, osimertinib demonstrated a concentration-dependent significant decrease of the resistance to paclitaxel, colchicine, and vincristine in KB-C2 cells (Table 1). In contrast, osimertinib at the concentration of 1.0 μM did not significantly increase the cytotoxicity of cisplatin, which is not a substrate of ABCB1 transporter. This reversal effect was similar to the one obtained when using verapamil at 3.0 µM or zosuquidar at 0.3 µM.

Furthermore, we used the ABCB1-transfected cell line HEK/ABCB1 and parental cell line HEK293/pcDNA3.1 to limit those factors to only one modulated by ABCB1 [26]. Similarly, osimertinib, at 0.3, 0.5, and 1.0 μM, produced a concentration-dependent decrease in ABCB1-mediated resistance to paclitaxel and vincristine (Table 2). However, osimertinib at 1.0 μM did not significantly alter the sensitivity of the empty vector transfected HEK293/pcDNA3.1 cells. We also used verapamil and zosuquidar as positive controls, and we obtained similar results. These results indicate that osimertinib could significantly reverse ABCB1-mediated MDR.

### 2.2. Effects of Osimertinib on Cell Lines Overexpressing ABCG2, ABCC1, or ABCC10

In order to determine the reversal effect of osimertinib on ABCG2-mediated MDR in ABCG2-overexpressing human cancer cells, we used the parental NCI-H460 cell line and the drug-selective NCI-H460/MX20 cell line. We found that osimertinib at 0.3 μM, a non-toxic drug concentration, significantly decreased the resistance of mitoxantrone in ABCG2 overexpressing NCI-H460/MX20 cells. However, osimertinib did not sensitize the parental NCI-H460 cells to mitoxantrone (Table 3).

Moreover, we also analyzed the effect of osimertinib on ABCC1- and ABCC10-mediated MDR. We found that osimertinib at 0.3 μM, a non-toxic drug concentration, had no significant reversal effect on ABCC1- and ABCC10-mediated MDR in ABCC1 overexpressing HEK/ABCC1 cells and ABCC10 overexpressing HEK/ABCC10 cells, respectively (Table 3).

Together these results indicate that osimertinib could reverse the ABCB1- and ABCG2-mediated MDR, but not ABCC1- and ABCC10-mediated MDR.

### 2.3. Effect of Osimertinib on the Intracellular Accumulation of [^3^H]-Paclitaxel

**Figure 2:**
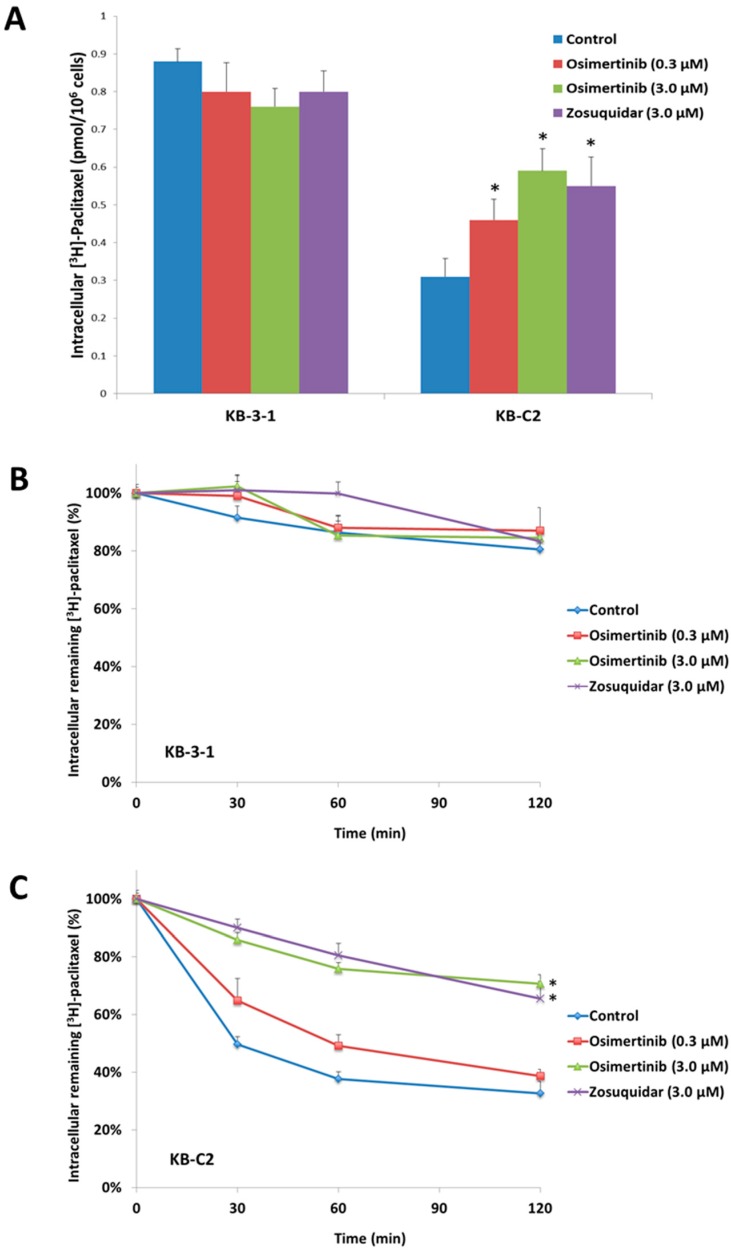
Effect of osimertinib on the accumulation and efflux time-course of [^3^H]-paclitaxel. (**A**) The accumulation of [^3^H]-paclitaxel was measured on parental KB-3-1 and ABCB1 overexpressing KB-C2 drug selected cell line. Columns are the mean of triplicate determinations; error bars represent SD; (**B**) time courses versus percentage of intracellular [^3^H]-paclitaxel remaining was plotted to show the effect of osimertinib in the KB-3-1 cell line; and (**C**) time courses versus percentage of intracellular [^3^H]-paclitaxel remaining was plotted to show effect of osimertinib in the KB-C2 cell lines. Lines are the mean of triplicate determinations; error bars represent SD. * *p* < 0.05 versus the control group. Experiments were performed at least three independent times.

To investigate the reversal mechanism, we studied the effect of osimertinib on the intracellular accumulation of [^3^H]-paclitaxel in ABCB1 overexpressing cells. We found that osimertinib at 0.3 and 3.0 μM produced a significant increase in the intracellular accumulation of [^3^H]-paclitaxel in KB-C2 cells (Figure 2A) while osimertinib did not alter the intracellular accumulation of [^3^H]-paclitaxel in the parental KB-3-1 cells. The effects were well comparable to that of zosuquidar (3 μM), a known inhibitor of ABCB1. These results suggested that osimertinib significantly increased intracellular concentrations of chemotherapeutic drugs in ABCB1-overexpressing cells and cause the increasing of cytotoxicity to these MDR cells.

### 2.4. Effect of Osimertinib on the Efflux of [^3^H]-Paclitaxel

We tested the efflux of [^3^H]-paclitaxel with or without osimertinib at different time points in ABCB1 overexpressing cells to determine if the increase in intracellular [^3^H]-paclitaxel accumulation caused by osimertinib was due to the inhibition of [^3^H]-paclitaxel efflux. [^3^H]-paclitaxel efflux occurred in the drug selective KB-C2 and parental KB-3-1 cells after removal of [^3^H]-paclitaxel from the culture medium. KB-3-1 cells lost 19.6% of the normalized intracellular [^3^H]-paclitaxel at the end of 2 h efflux (Figure 2B). Meanwhile, more than 67.0% loss of normalized [^3^H]-paclitaxel accumulation was observed in KB-C2 cells (Figure 2C). Treatment with osimertinib or ABCB1 inhibitor zosuquidar increased the retention of [^3^H]-paclitaxel in ABCB1-overexpressing cells. Effects of osimertinib followed a concentration-dependent pattern and 3.0 μM osimertinib exhibited 70.7% of [^3^H]-paclitaxel retention in KB-C2 cells. Neither osimertinib nor zosuquidar significantly altered efflux pattern in parental KB-3-1 cells.

### 2.5. Effect of Osimertinib on the Protein Expression and Location of ABCB1

**Figure 3:**
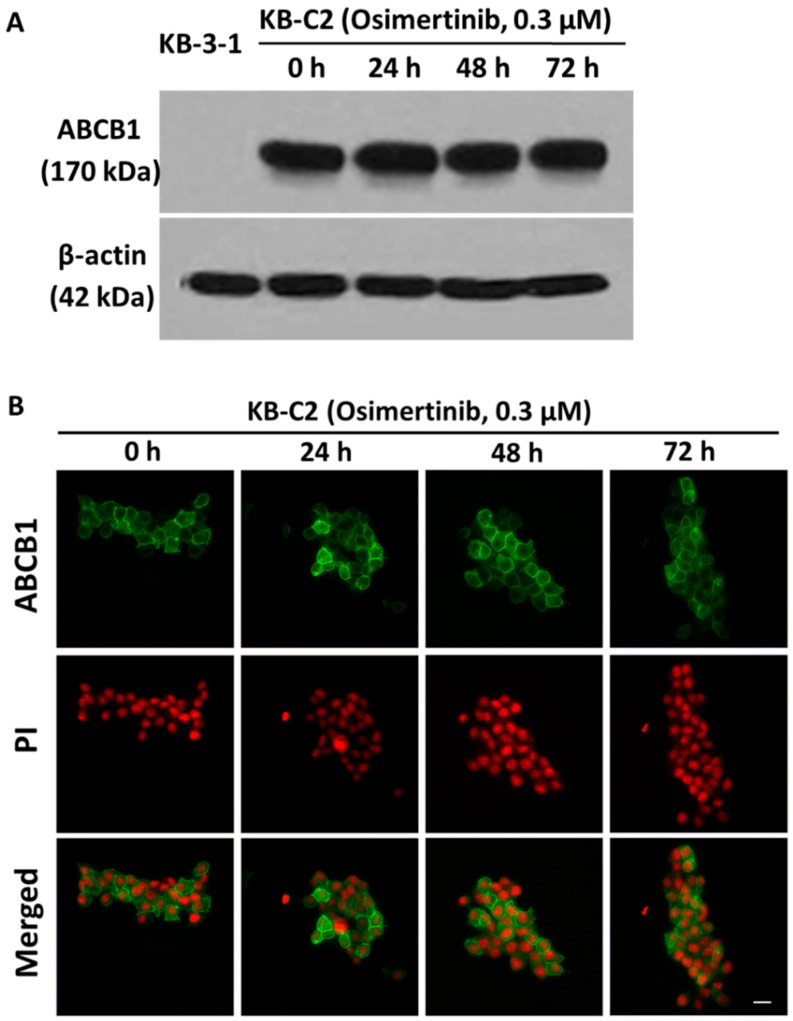
Effect of osimertinib on the protein expression and location of ABCB1. (**A**) The effect of osimertinib at 0.3 μM on the expression of ABCB1 was measured in KB-3-1 and KB-C2 cell lines for 0, 24, 48, and 72 h. Equal amounts of total cell lysate were used for each sample; and (**B**) the effect of osimertinib at 0.3 μM on the subcellular localization of ABCB1 was measured in ABCB1-overexpressing KB-C2 cells for 0, 24, 48, and 72 h. Scale bar, 10 μm. PI (propidium iodide, red) counterstains the nuclei. ABCB1 staining is shown in green.

The reversal of ABCB1-mediated MDR can be achieved either by decreasing ABCB1 expression at the cell surface or inhibiting the function of ABCB1 transporter. To analyze the effect of osimertinib on the ABCB1 expression, we performed a Western blot analysis of KB-3-1 cells and KB-C2 cells incubated with 3.0 μM osimertinib for 0, 24, 48, and 72 h. We found that there was no significant change in the expression of ABCB1 upon osimertinib treatment (Figure 3A). To analyze if osimertinib causes a translocation of ABCB1 from the plasma membrane to the cytoplasm, we performed an immunofluorescence analysis incubated KB-C2 cells with 3.0 μM osimertinib for 0, 24, 48, and 72 h. The results showed that the membrane expression and location of ABCB1 were not significantly changed (Figure 3B).

### 2.6. Interaction Analysis of MD-Stabilized Osimertinib-ABCB1 Complex

**Figure 4:**
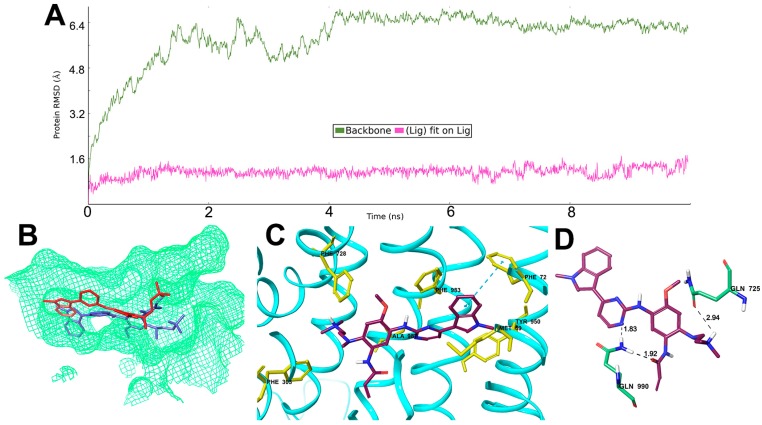
Molecular modeling of binding of osimertinib to homology ABCB1. (**A**) RMSD trajectory of ABCB1 and osimertinib in the ABCB1- osimertinib complex over the 10 ns simulation run; (**B**) change in position of osimertinib after simulation (red, post MD; blue, pre MD); (**C**) hydrophobic contacts found in ABCB1-osimertinib complex post MD simulation; and (**D**) hydrogen bond interactions and polar interactions between osimertinib and ABCB1 after MD simulation.

To study the dynamic nature of interactions, a molecular dynamics simulation was carried out for ABCB1 in complex with osimertinib for 10 ns. All protein frames are first aligned on the reference frame backbone, and then the root mean square deviation (RMSD) is calculated. Monitoring the RMSD of the protein can indicate if the simulation has equilibrated. The backbone of the protein in the complex deviated up to about 6.5 Å in the first 4 ns after which it acquired an almost stable conformation which then persisted until the end of simulation period (Figure 4A). The ligand, osimertinib, was stable after 1 ns of simulation with a RMSD up to 1.5 Å, indicating small internal fluctuations inside the binding pocket. The superimposition of the ligand osimertinib in the pre- and post-MD simulated complex structures inside the ligand binding site is depicted in Figure 4B. Hydrophobic interactions between ABCB1 and osimertinib involved residues Met69, Phe72, Phe303, Phe728, Tyr950, Phe983 and Ala987. The phenyl ring of Phe72 formed a π-π interaction with the 1-methylindol ring of osimertinib, which occurs more than 50% of the simulation time. Similarly, a π-cation interaction was formed between Phe728 and the dimethylamino nitrogen atom of osimertinib. These hydrophobic interactions were shown in Figure 4C. Post simulation, hydrogen bonds and polar interactions were found between osimertinib and residue Gln725 and Gln990 as shown in Figure 4D.

### 2.7. Effect of Osimertinib on the ABCB1 ATP Hydrolysis

**Figure 5:**
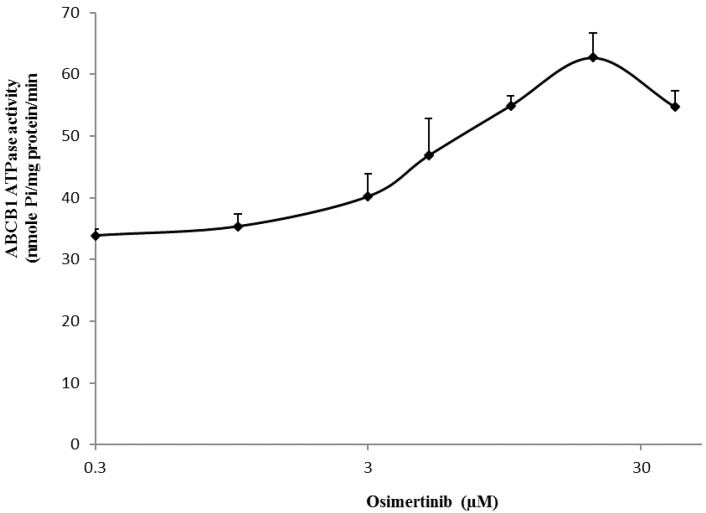
Effect of osimertinib on the Vi-sensitive ABCB1 ATPase activity. The Vi-sensitive ATPase activity of ABCB1 in membrane vesicles was determined with different concentrations of osimertinib as described in the “Materials and Methods” section. The mean values are plotted and error bars depict ± SD (*n* = 3).

The ABCB1 transporter utilizes energy derived from the hydrolysis of ATP to efflux their substrates across the membrane against a concentration gradient, thus ATP consumption reflects its ATPase activity [27]. To assess the effect of osimertinib on the ATPase activity of ABCB1, we measured the ABCB1-mediated ATP hydrolysis in the presence of osimertinib at various concentrations from 0–40 µM. Similar to other TKIs, osimertinib stimulated the ATPase activity of ABCB1 in a concentration-dependent manner, with a maximal stimulation of 2.61-fold of the basal activity (Figure 5). The concentration of osimertinib required to obtain 50% stimulation is 1.28 µM. The result indicated that osimertinib interacts at the drug-substrate-binding site and affect the ATPase activity of ABCB1.

## 3. Material and Methods

### 3.1. Chemicals

Osimertinib (chemical purity: >99.5%, HPLC at 214 and 254 nm) was purchased from ChemieTek (Indianapolis, IN, USA). Dulbecco’s modified Eagle’s Medium (DMEM), fetal bovine serum (FBS), penicillin/streptomycin and trypsin 0.25% were purchased from Hyclone (GE Healthcare Life Science, Pittsburgh, PA, USA). Bovine serum albumin (BSA), monoclonal antibody C219 (against ABCB1), monoclonal antibody BA3R (against beta-actin), Alexa Fluor 488 conjugated goat anti-mouse IgG secondary antibody were purchased from Thermo Fisher Scientific Inc. (Rockford, IL, USA). Cepharanthine was purchased from Apexbio Technology LLC (Houston, TX, USA). Fumitremorgin C (FTC) was provided by Dr. Susan E. Bates from NIH (Bethesda, MD, USA). Verapamil, zosuquidar, 3-(4,5-dimethylthiazol-yl)-2,5-diphenyltetrazolium bromide (MTT), colchicine, paclitaxel, vincristine, cisplatin, mitoxantrone, dimethylsulfoxide (DMSO), propidium iodide, ammonium molybdate, MES hydrate, antimony potassium tartrate, sodium azide, and *N*-methyl-d-glucamine, and other chemicals were products from Sigma-Aldrich (St. Louis, MO, USA). [^3^H]-paclitaxel (15 Ci/mmol) was purchased from Moravek Biochemicals, Inc (Brea, CA, USA). The chemicals used in ATPase assay were same as those in our previous study [28]. Potassium phosphate, EGTA and ATP were products of AMRESCO (Solon, OH, USA). Sulfuric acid solution (37 N) was purchased from Fisher Scientific (Pittsburgh, PA, USA). KCl was the product of Avantor Performance Materials (Center Valley, PA, USA). Ouabain was purchased from Enzo Life Sciences, Inc. (Farmingdale, NY, USA). Dithiothreitol was the product of Promega Corporation (Madison, WI, USA). MgCl_2_ was purchased from EMD Millipore (Billerica, MA, USA). Ascorbic acid was the product of VWR International (West Chester, PA, USA). Sodium orthovanadate was purchased from Alfa Aesar (Ward Hill, MA, USA). An OPSYS microplate reader was purchased from Dynex Technologies (Chantilly, VA, USA).

### 3.2. Cell Lines and Cell Culture

Human epidermoid carcinoma cell line KB-3-1 and its colchicine-selected ABCB1-overexpressing KB-C2 cell line, which survives increasing concentration of colchicine for up to 2 µg/mL, were used for the ABCB1 reversal study [27]. Non-small cell lung cancer cell line NCI-H460 and its mitoxantrone-selected ABCG2-overexpressing NCI-H460/MX20 cells, which was cultured with the addition of 20 nM mitoxantrone, were used in the ABCG2 reversal study [29]. HEK293/pcDNA3.1, HEK/ABCB1, HEK/ABCC1, and HEK/ABCC10 cells were generated by transfecting the HEK293 cells with empty pcDNA3.1 vector, ABCB1 expression vector, ABCC1 expression vector, and ABCC10 expression vector, and were cultured in medium with 2 mg/mL of G418 [30]. All cell lines were grown as adherent monolayers in DMEM supplemented with 10% fetal bovine serum (FBS), 1% penicillin/streptomycin in a 5% CO_2_ incubator at 37 °C. All cells were grown in drug-free culture medium for more than 15 days before assay.

### 3.3. Cytotoxicity by MTT Assay

Cytotoxicity and reversal experiments were performed using the MTT colorimetric assay as described previously [31,32]. Cells were harvested and resuspended in a final concentration of 4 × 10^3^ cells/well for the KB-3-1, KB-C2, NCI-H460, and NCI-H460/MX20 cells, and 6 × 10^3^ cells/well for the HEK 293/pcDNA3.1, HEK/ABCB1, HEK/ABCC1, and HEK/ABCC10 cells. Cells were seeded evenly into each well with 160 µL medium in 96-well microplates in triplicate and cultured at 37 °C. After 24 h of incubation, cells were pre-incubated with or without the reversal agents (20 µL/well) for 2 h. Then different concentrations of chemotherapeutic drugs (20 µL/well) were added into designated wells. After 72 h of incubation, 20 μL of MTT solution (4 mg/mL) was added into each well. The microplate was further incubated for an additional 4 h, and then the supernatant was discarded and 100 μL of DMSO were added to dissolve the formazan crystals. Finally, the absorbance was determined at 570 nm by the OPSYS microplate reader (Dynex Technology, Chantilly, VA, USA). All of the experiments were repeated at least three times, and the mean and standard deviation (SD) values were calculated.

### 3.4. [^3^H]-Paclitaxel Accumulation Assay

The accumulation of [^3^H]-paclitaxel in KB-3-1 and KB-C2 cells was measured in the presence or absence of inhibitors. Briefly, the cells (5 × 10^6^ cells) were resuspended and incubated in the medium in the presence or absence of osimertinib (0.3 and 3 μM) or zosuquidar (3 μM) at 37 °C for 2 h. Then, cells were incubated with 1 nM [^3^H]-paclitaxel containing medium for an additional 2 h at 37 °C, with or without osimertinib (0.3 and 3 μM) or zosuquidar (3 μM), and subsequently washed twice with ice-cold PBS. Then the cells were lysed and placed in 5 mL scintillation fluid and radioactivity was measured in the Packard TRI-CARB 1900CA liquid scintillation analyzer (Packard Instrument, Downers Grove, IL, USA) [33].

### 3.5. [^3^H]-Paclitaxel Efflux Assay

For the efflux assay, KB-3-1 and KB-C2 cells were incubated sequentially with medium with 1 nM [^3^H]-paclitaxel for 2 h, and then fresh drug-free medium for 2 h with or without osimertinib (0.3 and 3 μM) or zosuquidar (3 μM). After 0, 30, 60, and 120 min, the aliquots of cells were removed and immediately washed twice with ice-cold PBS [34]. Then the cells were lysed and placed in 5 mL scintillation fluid and radioactivity was measured in the Packard TRI-CARB 1900CA liquid scintillation analyzer (Packard Instrument, Downers Grove, IL, USA).

### 3.6. Western Blot Analysis

The KB-3-1 and KB-C2 cells were treated with or without reversal drugs for different time periods (0, 24, 48, 72 h), and cells were lysed after washing twice with ice-cold PBS. The cell extracts were prepared by incubating cells on ice for 20 min with lysis buffer (10 mM Tris HCl, pH 7.5, 1 mM EDTA, 0.1% SDS, 150 mM NaCl, 1% Triton X-100 and protease inhibitor cocktail). After centrifuging at 4 °C for 5 min, the supernatant which containing total cell lysate was collected and then protein concentration was determined by bicinchoninic acid (BCA^TM^)-based protein assay (Thermo Scientific, Rockford, IL, USA). Equal amounts of protein were resolved by sodium dodecyl sulfate polyacrylamide gel electrophoresis (SDS-PAGE) and transferred onto polyvinylidene fluoride (PVDF) membranes through electrophoresis. After blocking in 5% skim milk for 1 h, the PVDF membranes were immunoblotted overnight with primary monoclonal antibodies against actin at 1:1000 dilution or ABCB1 at 1:500 dilution at 4 °C, and were then further incubated for 2 h at room temperature with HRP (horseradish peroxidase)-conjugated secondary antibody (1:1000 dilution). The signal was detected using enhanced chemiluminescence detection system (Amersham, NJ, USA) [35].

### 3.7. Immunofluorescence

Cells (2 × 10^4^) were seeded in 24-well plates and were allowed to grow overnight, followed by treatment with 0.3 μM osimertinib for 0, 24, 48 and 72 h respectively. Then the cells were fixed in 4% paraformaldehyde for 15 min, permeabilized by 0.1% Triton X-100 for 10 min and then blocked with 6% BSA for 1.5 h at room temperature. Subsequently, cells were incubated with monoclonal antibody P7965 against ABCB1 (1:400) overnight, followed by Alexa Fluor 488 conjugated secondary antibody (1:1000) for 1 h. Propidium iodide solution was used to counterstain the nuclei. Immunofluorescence images were collected using a Nikon TE-2000S fluorescence microscope (Nikon Instruments Inc., Melville, NY, USA).

### 3.8. Induced-Fit Docking and Molecular Dynamics (MD) Simulation

Human ABCB1 homology model based on refined mouse ABCB1 (PDB ID: 4M1M) was kindly provided by S. Aller and the docking grid was refined as previously described [36]. The structure of osimertinib was built using builder panel in Maestro and ligand preparation was carried out for this compound by Ligprep 3.3 module (Schrödinger, Cambridge, MA, USA, 2015). The energy minimized compound was subjected to Glide v6.6 XP (extra precision) docking. As the protein is held rigid and the ligand is free to rotate, the simulation may provide misleading results. Also, the conformational changes of protein may allow the protein to generate close conformations to the shape of the ligand and lead to good binding affinity complex. In this study, the IFD (induced-fit docking) [37] was carried out using Glide v6.6 (Schrödinger, Cambridge, MA, USA, 2015). At the transmembrane site of ABCB1, the best scored osimertinib binding from XP run was used to generate the grid for IFD calculation. The default Glide IFD protocol was followed and the docking score (kcal/mol) was calculated.

The docked complex was then subjected to short MD simulation for validation. MD simulation was performed with the established program Desmond [38], in the NPT ensemble, with explicit TIP3P waters and counter-ions added to neutralized the overall charge of the system. The system was prepared in Maestro with the POPC bilayer placed. The default setup parameters were kept and periodic boundary conditions were applied. The distance between box wall and ABCB1-osimertinib complex was set to greater than 10 Å so that the complex does not directly interact with its own periodic image. The MD was performed at constant temperature (300 K) and pressure (1.015 bar) using a time step of 2 fs, and coordinates saved every 5 ps. The OPLS 2005 force-field was used. Desmond v4.5’s default protocol was applied to equilibrate the system. The equilibrated complex system was then subjected to 10,000 ps (10 ns) MD simulation.

The root mean square deviation (RMSD) of the protein-ligand complex was calculated for the entire simulations trajectory using the first frame as reference. Schrödinger simulation interactions diagram (SID) was used to analysis the interactions.

### 3.9. ABCB1 ATPase Activity Assay

The vanadate-sensitive ATPase activity of ABCB1 measured in the membrane vesicles obtained from BD Biosciences (San Jose, CA, USA). The membrane vesicles (20 µg protein/reaction) were incubated in ATPase assay buffer at 37 °C for 5 min with or without 400 µM vanadate. The membrane vesicles were incubated with different concentrations of osimertinib, ranging from 0–40 µM, at 37 °C for 5 min, followed by an addition of 10 µL of 25 mM Mg-ATP solution. The reaction was allowed to continue for another 20 min at 37 °C, and then terminated by an addition of 100 µL 5% SDS solution to the reaction mix. The amount of Pi release was detected and quantified by adding 200 µL of detection reagent. The reaction mix then further incubated for 10 min at room temperature. The absorption was detected at 880 nm using a spectrophotometer.

### 3.10. Statistical Analysis

All experiments were repeated at least three times and the differences were determined by using the one-way ANOVA followed by Newman-Keuls post hoc test for comparing multiple groups with one variable in the following experiments: cell viability assay, accumulation assay. Statistical analysis was performed by two-way ANOVA followed by Bonferroni post hoc test for comparing multiple groups with more than one variable in the following experiments: efflux assay. Differences were considered significant when *p* < 0.05.

## 4. Discussion

Previous studies have shown several small molecule cell signaling inhibitors, such as tyrosine kinase inhibitors, play an important role in drug resistance in clinical medicine. Many clinically-used TKIs significantly reverse ABC transporter-mediated MDR. About 85% of lung cancers are NSCLC. Approximately 15% of patients with advanced NSCLC have tumors that are EGFR mutated. When the EGFR on the surface of cells is mutated and becomes active, it can lead to cancer growth [22]. Most advanced lung cancers eventually progress because the tumors become resistant to TKI therapy. In patients who have EGFR mutated NSCLC and progressed on certain EGFR TKI therapies, 67% of cases had the T790M mutation and which is related to the progression of the disease. T790M is a mutation that develops in response to first-line treatment of the EGFR mutation [21]. Several reports have shown the expression of high levels of ABCC1 and ABCC3 in lung cancer specimens. A high incidence of ABCC1 expression in NSCLC has been reported compared to small cell lung cancer (SCLC). In some lung cancer specimens, other ABC transporters, such as ABCB1 and ABCG2, were detected in low quantities. In addition to the previously mentioned ABC transporters ABCB1, ABCG2, and ABCC1, ABCC10 expression may be considered as a predictive biomarker for MDR in non-small cell lung cancer treatment following treatment with paclitaxel [6]. Osimertinib is an oral, irreversible, and mutant-selective EGFR TKI in treating patients with metastatic EGFR T790M-positive tumors. Since many cancer cells may develop MDR via different types of ABC transporters, we examined ABCB1, ABCG2, ABCC1, and ABCC10 in this study.

In this study, we used the first-generation reversal agent verapamil as a positive control reversal agent for the ABCB1-mediated MDR. The first-generation agents were always used in high doses since they did not selectively inhibit ABCB1 [39]. Therefore, they showed high toxicity, such as cardiac toxicity was observed with verapamil when used to overcome MDR. Second-generation inhibitors such as valspodar were less toxic and more potent than first-generation drugs. However, these inhibitors always cause unpredictable absorption and metabolism with many antineoplastic drugs due to also being substrates of enzymes, such as CYP 3A4 [40]. The third-generation inhibitors with high affinity, low toxicity, and enhanced selectivity were designed to overcome the side effects and unpredictable pharmacokinetic outcomes [41]. We also used third-generation agent zosuquidar for this study. Osimertinib at 1 µM significantly decreased the resistance of paclitaxel and vincristine in KB-C2 cells, and this reversal effect was better than the one obtained when using verapamil at 3 µM, but not as good as the one obtained when using zosuquidar at 0.3 µM.

This study reports that osimertinib can reverse ABCB1-mediated MDR. The cytotoxicity assay indicated that osimertinib significantly enhanced the cytotoxicity of ABCB1 substrates, such as paclitaxel and vincristine, in both drug selected KB-C2 and transfected HEK/ABCB1 cells. However, osimertinib up to 1 µM did not significantly sensitize the parental KB-3-1 and HEK293/pcDNA3.1 cells to the anticancer drugs used in this study. In addition, there was no significant alteration insensitivity of cancer cells to compounds that were not ABCB1 or ABCG2 substrates, which suggests that the efficacy of osimertinib to reverse MDR is specific to ABCB1 and ABCG2. Furthermore, drug accumulation studies demonstrated that osimertinib significantly enhanced the intracellular accumulation of [^3^H]-paclitaxel in KB-C2 cells overexpressing ABCB1 (Figure 2A). Molecular docking is a tool that can calculate the binding affinity of a protein-ligand complex. MD simulation is a technique for the calculation of “movement” of the complex. Several previous studies have revealed the problem that some well-docked ligand might “fly” away from the binding pocket in MD simulation [42]. In this study, we use MD simulation as an essential validation before we draw conclusions from docking results. Our results indicated that osimertinib strongly bind to the transmembrane drug-binding domain of ABCB1, and the low ligand RMSD showed that the binding pose is very stable through a 10 ns MD simulation (Figure 4A,B). Both hydrogen bonding interactions and hydrophobic interactions contributed to the binding of osimertinib. Additionally, osimertinib stimulated the ATPase activity of ABCB1 in a concentration-dependent fashion, with a maximal stimulation of 2.61-fold of the basal activity, indicating that osimertinib might be a competitive substrate of the ABCB1 transporter (Figure 5). These results were consistent with the cytotoxic results that osimertinib interacts with ABCB1 substrates and sensitizes ABCB1-mediated MDR cells to anticancer drugs. It is possible that the effect produced by osimertinib may be due to down-regulation of ABCB1 expression or translocation of ABCB1 from the plasma membrane to cytoplasm. However, the Western blot and immunofluorescence analysis results suggested that there were no significant changes in protein expression and localization of ABCB1. Therefore, we suggest that the reversal effect of osimertinib on ABCB1 in MDR cells is not due to its effect on ABCB1 expressional levels, but related to its inhibition of efflux and transport function. In the future study, we will find a way to directly show the link between cell viability and transporter activity if it is possible.

In conclusion, the reversal of ABCB1-mediated MDR by osimertinib involved the inhibition of ABCB1 efflux function without interfering ABCB1 protein expression and, thus, increased the intracellular accumulation of certain substrates into the MDR cells. Collectively, this study implied that the clinical response of conventional chemotherapeutic drugs to MDR could be improved when combined with osimertinib.

## Figures and Tables

**Table 1:** Reversal effects of osimertinib on ABCB1-mediated MDR in KB-3-1 and KB-C2 cell lines.

Treatment	KB-3-1		KB-C2	
	IC_50_ ± SD ^a^ (nM)	RF ^b^	IC_50_ ± SD (µM)	RF
Paclitaxel	3.58 ± 0.35	[1.0]	1.075 ± 0.299	[307.1]
+ Osimertinib (0.3 µM)	3.63 ± 0.49	[1.0]	0.603 ± 0.045 *	[172.3]
+ Osimertinib (0.5 µM)	3.57 ± 0.41	[1.0]	0.092 ± 0.008 *	[26.3]
+ Osimertinib (1.0 µM)	3.12 ± 0.47	[0.9]	0.017 ± 0.005 *	[4.9]
+ Verapamil (3.0 µM)	3.24 ± 0.44	[0.9]	0.056 ± 0.011 *	[16.0]
+ Zosuquidar (0.3 µM)	2.81 ± 0.36	[0.8]	0.013 ± 0.003 *	[3.7]
Colchicine	59.82 ± 8.57	[1.0]	15.528 ± 4.565	[259.6]
+ Osimertinib (0.3 µM)	56.60 ± 7.13	[0.9]	5.120 ± 0.982 *	[85.6]
+ Osimertinib (0.5 µM)	58.66 ± 5.20	[1.0]	1.279 ± 0.606 *	[21.4]
+ Osimertinib (1.0 µM)	52.53 ± 7.39	[0.9]	0.637 ± 0.071 *	[10.6]
+ Verapamil (3.0 µM)	58.87 ± 9.65	[1.0]	0.279 ± 0.015 *	[4.7]
+ Zosuquidar (0.3 µM)	52.54 ± 9.12	[0.9]	0.078 ± 0.020 *	[3.7]
Vincristine	5.05 ± 0.89	[1.0]	0.797 ± 0.019	[157.8]
+ Osimertinib (0.3 µM)	5.49 ± 0.54	[1.1]	0.789 ± 0.027	[156.3]
+ Osimertinib (0.5 µM)	5.26 ± 0.71	[1.0]	0.228 ± 0.037 *	[45.1]
+ Osimertinib (1.0 µM)	5.56 ± 0.96	[1.1]	0.027 ± 0.006 *	[5.2]
+ Verapamil (3.0 µM)	4.61 ± 0.86	[0.9]	0.062 ± 0.008 *	[12.3]
+ Zosuquidar (0.3 µM)	4.58 ± 0.65	[0.9]	0.013 ± 0.003 *	[2.6]
	IC50 ± SD (µM)	RF	IC50 ± SD (µM)	RF
Cisplatin	2.90 ± 0.27	[1.0]	3.12 ± 0.20	[1.1]
+ Osimertinib (1.0 µM)	2.78 ± 0.17	[1.0]	2.98 ± 0.22	[1.0]
+ Verapamil (3.0 µM)	2.61 ± 0.20	[0.9]	2.66 ± 0.31	[0.9]

^a^ IC_50_ values are represented as mean ± SD of at least three independent experiments performed in triplicate; ^b^ Values represent the resistance fold (RF) obtained by dividing IC_50_ value of anticancer drug in KB-3-1 and KB-C2 cells with or without reversal agent divided by the IC_50_ value of the respective anticancer drug in KB-3-1 cells without reversal agent. * *p* < 0.01 versus the control group.

**Table 2:** Reversal effects of osimertinib on ABCB1-mediated MDR in HEK293/pcDNA3.1 and HEK/ABCB1 cell lines.

Treatment	HEK293/pcDNA3.1		HEK/ABCB1	
	IC50 ± SD ^a^ (nM)	RF ^b^	IC50 ± SD (nM)	RF
Paclitaxel	26.88 ± 2.74	[1.0]	1920.76± 150.32	[71.5]
+ Osimertinib (0.3 µM)	27.53 ± 1.96	[1.0]	1497.33 ± 149.65	[55.7]
+ Osimertinib (0.5 µM)	28.46 ± 3.35	[1.1]	906.75± 58.96 *	[33.7]
+ Osimertinib (1.0 µM)	30.45 ± 2.80	[1.1]	200.31 ± 22.04 *	[7.5]
+ Verapamil (3.0 µM)	23.30 ± 3.11	[0.9]	85.79 ± 5.86 *	[3.2]
+ Zosuquidar (0.3 µM)	29.50 ± 2.87	[1.1]	35.54 ± 5.77 *	[1.3]
Vincristine	15.98 ± 2.88	[1.0]	566.19 ± 52.31	[35.4]
+ Osimertinib (0.3 µM)	16.98 ± 2.34	[1.1]	434.10 ± 50.89	[27.2]
+ Osimertinib (0.5 µM)	16.99 ± 1.96	[1.1]	269.55 ± 46.04 *	[16.9]
+ Osimertinib (1.0 µM)	15.22 ± 1.44	[1.0]	99.38 ± 38.92 *	[6.2]
+ Verapamil (3.0 µM)	14.84 ± 1.60	[0.9]	75.25 ± 27.55 *	[4.7]
+ Zosuquidar (0.3 µM)	13.26 ± 1.21	[0.8]	50.66 ± 20.54 *	[3.2]
Cisplatin	1092.52 ± 100.26	[1.0]	1193.56 ± 111.2	[1.0]
+ Osimertinib (1.0 µM)	985.27 ± 103.67	[0.9]	1011.04 ± 86.29	[1.1]
+ Verapamil (3.0 µM)	900.79 ± 83.54	[0.8]	1121.36 ± 114.73	[1.0]

^a^ IC_50_ values are represented as mean ± SD of at least three independent experiments performed in triplicate; ^b^ Values represent the resistance fold (RF) obtained by dividing IC_50_ value of anticancer drug in HEK293/pcDNA3.1 and HEK/ABCB1 cells with or without reversal agent divided by the IC_50_ value of the respective anticancer drug in HEK293/pcDNA3.1 cells without reversal agent. * *p* < 0.01 versus the control group.

**Table 3:** Effects of osimertinib on ABCG2-, ABCC1-, and ABCC10-mediated MDR in parental and resistant cell lines.

Treatment	NCI-H460		NCI-H460/MX20	
	IC50 ± SD ^a^ (µM)	RF ^b^	IC50 ± SD (µM)	RF
Mitoxantrone	0.13 ± 0.05	[1.0]	24.97 ± 5.46	[193.86]
+ Osimertinib (0.3 µM)	0.15 ± 0.05	[1.2]	10.44 ± 5.92 *	[80.31]
+ FTC (3 µM)	0.12 ± 0.07	[0.9]	0.57 ± 0.11 *	[4.36]
Treatment	HEK293/pcDNA3.1		HEK/ABCC1	
	IC50 ± SD (nM)	RF	IC50 ± SD (nM)	RF
Vincristine	9.12 ± 1.12	[1.0]	91.74 ± 5.23	[10.1]
+ Osimertinib (0.3 µM)	8.59 ± 1.57	[0.9]	72.96 ± 9.65	[8.0]
+ MK571 (25 µM)	8.95 ± 1.48	[1.0]	9.86 ± 2.02 *	[1.1]
Treatment	HEK293/pcDNA3.1		HEK/ABCC10	
	IC50 ± SD (nM)	RF	IC50 ± SD (nM)	RF
Paclitaxel	13.45 ± 1.03	[1.0]	172.61± 8.99	[12.8]
+ Osimertinib (0.3 µM)	14.48 ± 1.38	[1.1]	146.26 ± 14.21	[10.9]
+ Cepharanthine (3 µM)	11.96 ± 1.26	[0.9]	21.15 ± 5.87 *	[1.6]

^a^ IC_50_ values are represented as mean ± SD of at least three independent experiments performed in triplicate; ^b^ Values represent the resistance fold (RF) obtained by dividing IC_50_ value of anticancer drug in NCI-H460 and NCI-H460/MX20 cells with or without reversal agent divided by the IC_50_ value of respective anticancer drug in NCI-H460 cells without reversal agent. The RF for HEK293/pcDNA3.1, HEK/ABCC1 and HEK/ABCC10 cells were obtained in a similar manner. * *p* < 0.01 versus the control group.
